# Long-term effects of device-guided slow breathing in stable heart failure patients with reduced ejection fraction

**DOI:** 10.1007/s00392-018-1310-7

**Published:** 2018-06-25

**Authors:** Kamila Lachowska, Jerzy Bellwon, Krzysztof Narkiewicz, Marcin Gruchała, Dagmara Hering

**Affiliations:** 1 1st Department of Cardiology, Medical University of Gdansk, Gdańsk, Poland; 2 Department of Hypertension and Diabetology, Medical University of Gdansk, Debinki 7c, 80-952 Gdańsk, Poland

**Keywords:** Heart failure with reduced ejection fraction, Slow breathing, Hemodynamics, 6-Min walk test, Functional capacity, Heart rate variability

## Abstract

**Background:**

Slow breathing (SLOWB) alleviates symptoms of chronic heart failure (HF) but its long-term effects are unknown. We examined the acute and long-term impact of device-guided breathing on hemodynamics and prognostic parameters in HF patients with reduced ejection fraction (HFrEF).

**Methods and results:**

Twenty-one patients with HFrEF (23.9 ± 5.8%, SD ± mean) on optimal medical therapy underwent blood pressure (BP), heart rate (HR), HR variability, 6-min walk test (6MWT), cardiopulmonary exercise testing (CPET), and echocardiography measurements before and 3 months after SLOWB home training (30 min daily). After 3 months, all patients were assigned to continue SLOWB (Group 1) or no-SLOWB (Group 2). All tests were repeated after 6 months. Acute SLOWB (18 ± 5 vs 8 ± 2 breaths/min, *P* < 0.001) had no influence on BP and HR but improved saturation (97 ± 2 vs 98 ± 2%, *P* = 0.01). Long-term SLOWB reduced office systolic BP (*P* < 0.001) but not central or ambulatory systolic BP. SLOWB reduced SDNN/RMSSD ratio (*P* < 0.05) after 3 months. One-way repeated measures of ANOVA revealed a significant increase in 6MWT and peak RER (respiratory exchange ratio) from baseline to 6-month follow-up in group 1 (*P* < 0.05) but not group 2 (*P* = 0.85 for 6MWT, *P* = 0.69 for RER). No significant changes in echocardiography were noted at follow-up. No HF worsening, rehospitalisation or death occurred in group 1 out to 6-month follow-up. Two hospitalizations for HF decompensation and two deaths ensued in group 2 between 3- and 6-month follow-up.

**Conclusions:**

SLOWB training improves cardiorespiratory capacity and appears to slow the progression of HFrEF. Further long-term outcome studies are required to confirm the benefits of paced breathing in HFrEF.

## Introduction

Chronic heart failure (HF) remains a challenging problem with a considerable impact on the global burden of cardiovascular (CV) morbidity and mortality [1–3]. Despite advances in HF prevention and management, the worldwide prevalence of newly diagnosed patients with HF is expected to rise further, accounting for a 46% increase in prevalence from 2012 to 2030 [4–9]. This is driven by prolonged life expectancy, improvements in therapies for coronary artery disease (CAD) and sudden cardiac death, and the growing incidence of co-morbidities (i.e. hypertension, diabetes) contributing to the development of HF [10–16]. Differentiation of patients with HF is critical due to diverse underlying aetiologies, associated co-morbidities and responses to treatment [17–23]. Pharmacological therapies have improved survival and reduced hospital admission in HF [24–27]. However, hypotension and resulting tachycardia often prevent further drug initiation and up-titration. Along with pharmacological approaches, surgical implantable electrical devices for the treatment of HF patients with reduced ejection fraction (HFrEF) improve symptoms, reduce the risk of death and all-cause mortality in primary and secondary prevention [10, 28–30]. Nevertheless, in HFrEF patient outcomes remain unsatisfactory high with an increased risk for sudden death, worsening HF, frequent hospitalization for CV events and recurrent decompensation [31–33]. Given that currently available optimal medical drug and device therapies are insufficient to halt disease progression, an unmet need for other therapeutic approaches clearly exists [34–38].

Amongst behavioural interventions, slowing spontaneous breathing rate below 10 breaths/min has the potential to favourably affect CV regulation [39–42]. The use of slow breathing (SLOWB) technique has been shown to reduce dyspnoea, improve oxygen saturation and exercise tolerance in HF patients, acutely increase baroreflex gain and stability in patients with CV disease and a risk for sudden death [42–45]. Data from pilot studies of patients with systolic chronic HF have demonstrated the feasibility of device-guided SLOWB pacing with the use of the RESPeRATE, improvements in NYHA class and left ventricle ejection fraction (LVEF), reductions in pulmonary pressure [46, 47] and breathlessness [48]. The effects of SLOWB training on blood pressure (BP) in chronic HF has been reported to be marginal with low incidence of orthostatic hypotension [49]. A recent study has demonstrated an improvement of physical capacity and systolic heart function with a tendency to attenuate sleep disturbances in chronic HF [50]. Although the currently available results with paced breathing are promising in chronic HF and the mechanistic rationale for the use of SLOWB is apparent, not all HF patients seemed to respond to this behavioural technique [48]. Previous studies in HF were limited to acute effects of SLOWB or 10–12 weeks in duration. The long-term impact of regular SLOWB performance on prognostic factors in chronic HF has not yet been investigated. Therefore, this study sought to comprehensively explore the effects of home-paced breathing on clinical, hemodynamic and prognostic parameters in stable patients with severe HFrEF, all of whom received optimal medical drug and device-based therapies.

## Methods

### Subjects

**Fig. 1:**
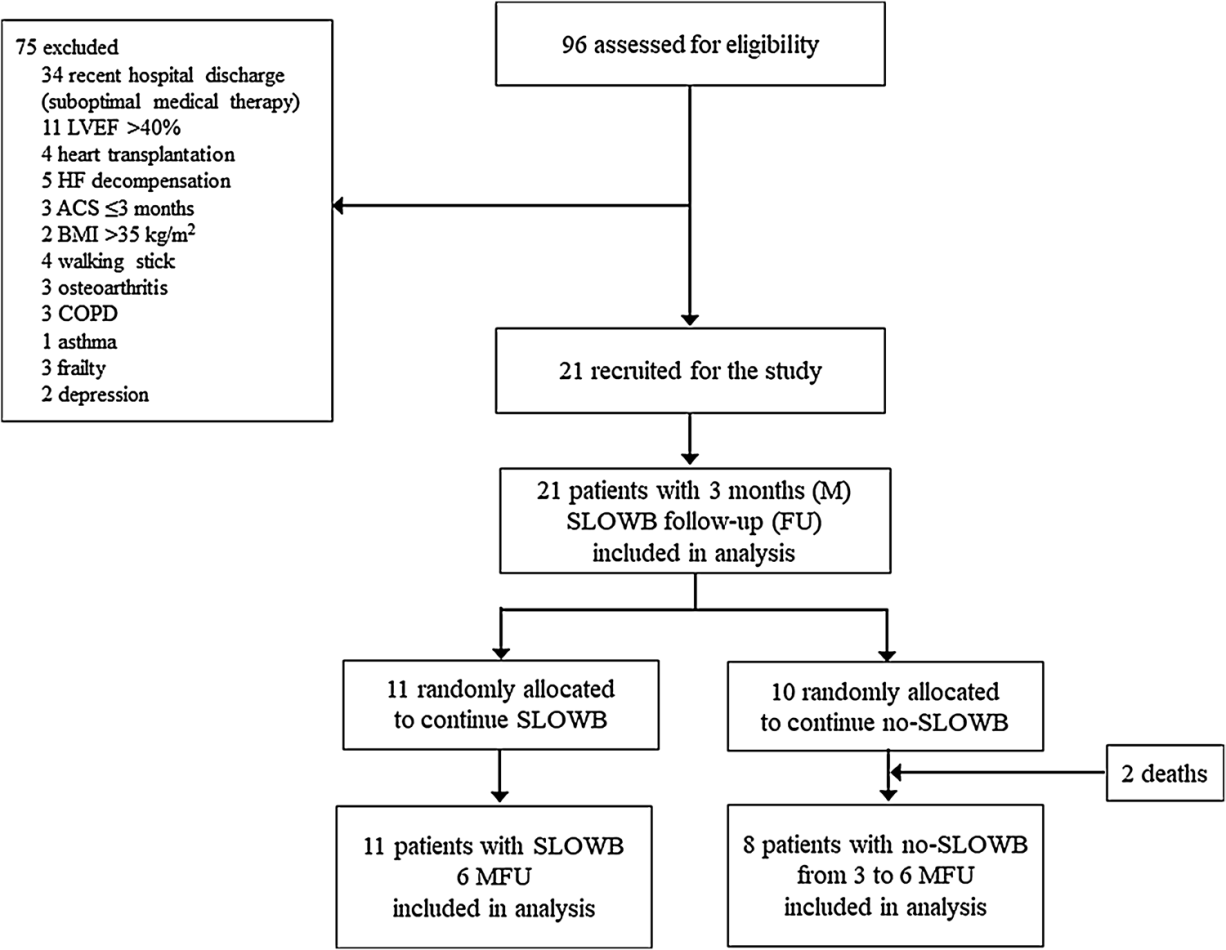
Patient flow diagram. Study patient recruitment flow chart based on CONSORT guidelines

This prospective unblinded case-series study was approved by the Institutional Ethics Committee and written informed consent was obtained from all patients. Eligible participants were adults aged 18 or over who met the eligibility criteria for HFrEF diagnosis and managements as per European Society of Cardiology (ESC) guidelines [10, 51]. Only stable patients with chronic HFrEF who were receiving optimal medical pharmacological (i.e. maximum tolerated dose of all recommended drug classes) and surgical implementable device therapies were recruited into the study. The inclusion criteria required stable unchanged medication (without a need to increase a dose of furosemide) for at least 6 weeks prior to study enrolment. Exclusion criteria were acute coronary syndrome (ACS) ≤ 3 months, percutaneous coronary angioplasty ≤ 3 months, coronary artery bypass graft ≤ 3 months, acute cerebrovascular disease ≤ 3 months, chronic obstructive pulmonary disease, asthma, upper pulmonary tract infection, medication nonadherence, depression, alcoholism, and the lack of patient’s cooperation. Study patient recruitment flow chart is shown in Fig. 1.

### Study settings

The study took place at the Medical University of Gdansk and the associated University Clinical Centre where patients were recruited from outpatient Cardiology Clinic. Comprehensive laboratory tests were conducted at the inpatient Cardiology Department and Department of Hypertension and Diabetology. This study took place from January 2015 to June 2017.

### Study protocol

All patients were hospitalized for three consecutive days at baseline, 3- and 6-month follow-up. During each study visit, all participants underwent comparable comprehensive tests. At the first day, following hospital admission, medical history, physical examination, anthropometric measurements (i.e. weight, height, waist and hip circumference), blood sampling and 6MWT were performed. Patients were instructed on how to use RESPeRATE^®^ device followed by the assessment of acute effects of SLOWB on continuous non-invasive BP, HR, respiration and saturation. Then, all patients were fitted with 24-h BP monitoring (ABPM) and 24-h Holter electrocardiogram (ECG). On the second day, patients underwent echocardiography measurements and cardiopulmonary exercise testing (CPET). Patients with implantable cardioverter-defibrillator (ICD) underwent intervention threshold check-up. On the third day of hospitalization, following the measurements of pulse wave velocity (PWV) and the assessment of the use of RESPeRATE^®^ device patients were discharged home. All patients were again hospitalized after 3 and 6 months to repeat all comparable measurements and clinical assessment.

### Office and ambulatory blood pressure

Automated office seated systolic BP (SBP) and diastolic BP (DBP) were measured simultaneously on both arms after at least 5 min of rest and was calculated as the average of three consecutive measurements within a 1-min interval using a validated Microlife^®^ WatchBP^®^ device which allows for dual-cuff simultaneous inter-arm assessment. The arm with higher BP readings was used for the subsequent assessment at 3- and 6-month follow-up.

24-h ABPM was performed with the Spacelabs 90207 recorder (Spacelabs Healthcare, Washington, United States) at baseline, 3- and 6-month follow-up as described previously [52].

### 24-h Holter ECG

24-h ECG recordings were performed at each study visit using three-channel Lifecard CF digital recorder (Del Mar Reynolds Medical Pathfinder SL 9.03.5910 software with 128 Hz sampling rate). Detailed manual analysis was performed by the same person (KL) to verify and correct all sinus and ectopic excitations including ventricular, supraventricular and artefacts. Heart rate variability (HRV) was assessed using time domain method for two of the 6-h periods corresponding to daytime and nighttime periods with sinus rhythm of at least 90%. The number of ectopic excitations, including ventricular and supraventricular extrasystoles, and artefacts was less than 10%. The following time domains were analysed: RR interval, SDNN (standard deviation of normal to normal R–R intervals), RMSSD (square root of the mean squared differences between successive RR intervals) as described previously [53].

### Transthoracic echocardiography

Echocardiographic ultrasound images were obtained in patent’s left lateral decubitus position using the GE VIVID S6 device (GE Medical Systems, Poland) with simultaneous ECG recordings as per standardized protocol [54, 55]. Echocardiographic measurements included left ventricle (LV) end-diastolic diameter (LVEDD), LV end-systolic diameter (LVESD), interventricular septum thickness (IVS), posterior wall thickness (PWT) in an end-diastolic period, left atrium (LA), LA area (LAA), left ventricle (LV) and calculated parameters including LV end-diastolic volume (LVEDV), LV end-systolic volume (LVESV) and LV ejection fraction (LVEF) in an apical four-chamber view (Simpson’s method). LV diastolic function was obtained by collecting of peak velocity of early rapid filling (E) in an apical four-chamber view. The Doppler sample was placed at the tips of opened mitral leaflets. Tissue Doppler imaging (TDI) of the mitral annulus in an apical four-chamber view included the early filling (*E*′) wave velocity. *E*/*E*′ ratio was also calculated. Echocardiographic assessment of the right heart included right ventricle (RV) and right atrial (RA) size measured from a four-chamber view obtained from the apical window at end-diastole. Measure of RV systolic function was assessed by tricuspid annular plane systolic excursion (TAPSE).

### Six-minute walk test (6MWT)

6MWT was performed at each study visit by an experienced physiotherapist. All patients received instruction on how to conduct the test, and written informed consent was obtained prior to test. Patients were advised to refrain from exercising 2 h before 6MWT. Following a 10-min rest in a sitting position, 6MWT was performed. The length of the corridor for the 6MWT was 30 m and was marked every 3 m to facilitate the calculation of the walking distance. The ends of the hallway, the start and return points were also marked. At the time of the test, patients marched on their own, at a comfortable and convenient pace, on a flat, hard surface of the empty hospital hallway. Twice during the test, patients were orally motivated to perform the exercise, during which current information about the test stage was given. As a result of the significantly reduced exercise tolerance, patients were allowed for short-term, patient-dependent pauses. Patients were monitored by telemetry during the 6MWT. In case of symptoms such as chest pain, sudden or severe dyspnoea, imbalance, fatigue or paleness, the 6MWT was discontinued [56].

### Cardiopulmonary exercise testing (CPET)

Each patient underwent CPET three times over the duration of the study (at baseline, 3- and 6-month follow-up) using the Ergocard Exercise Testing System (Medisoft, Belgium). Patients were asked not to perform intensive exercise for at least 48 h prior to test. The CPET System consists of cycle ergometer, the HR chest belt, 12-lead ECG module integrated with interface, spirometry and automated BP measurements. Each patient received clear instructions regarding the CPET performance and additional written consent was obtained prior to test. Following a calibration of the system, the assessment of exercise capacity was conducted on a stationary cycle ergometer where a subject was asked to maintain pedal speed at the level of 60–65 rev per minute. Electrocardiographic HR monitoring with multiple lead wave-forms was recorded during the entire exercise test and over a 3-min post-recovery period. BP was measured periodically every 3 min throughout the test. Symptoms including perceived exertion and dyspnoea were assessed and quantified immediately after the test completion (at the beginning of the recovery period). Functional exercise capacity was assessed using the RAMP exercise protocol including a 2-min warm-up phase and a modest increase in work rate (10 W per minute) until patients reached the maximum tolerance for their symptoms. ICD settings were checked prior to test. The peak HR during the test was kept below HR at which the ICD was programmed to discharge.

The following parameters were analyzed: (1) peak oxygen content P*V*O_2_ (ml/kg/min), the maximum rate of oxygen consumption measured during incremental exercise (indicative of maximal aerobic capacity), (2) peak RER (respiratory/gas exchange ratio), the ratio of carbon dioxide (*V*co_2_) output to oxygen (*V*O_2_) uptake at maximal exercise levels and (3) minute ventilation (VE) and Vco_2_ output slope as previously described [57–59].

### Pulse wave velocity

The measurements were performed in fasting condition, in a quiet room and supine position after 15 min of rest between 9.00 and 11.00 am and room temperature 21–23 °C as per Expert Consensus recommendation [60, 61]. After 15 min of rest, brachial BP was measured simultaneously in both arms which allow for dual-cuff simultaneous inter-arm assessment and was calculated as the average of three consecutive measurements within a 1-min interval. Following BP measurements, carotid–femoral (c–f) pulse wave velocity (PWV) was performed using an automatic Complior System (Del Mar Reynolds Medical). An online pulse wave recording and automatic calculation of PWV was conducted using two mechanotransducers, the first positioned at the base of the neck of the common carotid artery and the second over the femoral artery. The operator identified the accurate shape of recorded arterial waves by positioning the probe over the femoral artery. The transit time was determined by means of a correlation algorithm between each simultaneous recorded wave [21, 62].

### Serum biochemistry

Routine blood tests were performed in all patients at each study visits at the associated Clinical Laboratory Centre.

### Interventions

#### Acute effects of slow breathing

On the first day, prior to 24-h ABPM and Holter ECG, patients underwent baseline continuous beat-to-beat measurements of BP, HR, respiration and saturation (ADInstruments, Dual Bio Amp; ADInstruments, Ltd. Oxford, UK) over a 20-min duration followed by a 15-min duration of SLOWB with the use of RESPeRATE device.

#### Decompensated chronic HF

Acute decompensation of chronic HF was defined as acute or gradual exacerbation of clinical signs and symptoms of congestion (i.e. dyspnoea, water retention, ankle oedema, pulmonary wheezes or rales) that required additional immediate therapy (i.e. intravenous furosemide) and/or hospital admission.

#### Slow-breathing technique

A device-guided SLOWB pacing (goal below 10 breaths/min) was performed twice daily with each session lasting 15 min, through use of an ad hoc device RESPeRATE^®^ (Intercure Ltd. Northern Industrial Area, Israel) as described previously [63]. At the first visit, patients were instructed on how to synchronize their breathing with guiding tones generated by the RESPeRATE^®^ in response to their breathing pattern. Following the completion of all comprehensive tests at baseline, all patients received the device and a translated training manual on how to use it in the home setting. Performance of SLOWB exercise was scheduled for two 15-min daily sessions (in total 30 min per day) over the first 3 months. Patients were asked to breathe effortlessly and gradually at home, irrespective of the time of the day, in a quiet room and in a comfortable position as recommended in the RESPeRATE^®^ manual. All patients were called by an investigator (KL) weekly through the duration of the study to obtain information stored on a device regarding weekly duration of SLOWB exercise (i.e. number of session, therapeutic minutes, initial breathing, final breathing rate, the ability to synchronize respiratory rate with guiding tones, breath detection).

All patients were asked to repeat all tests after 3 months of SLOWB home exercise during a 3-day hospitalization.

#### Randomisation

At 3-month follow-up, using simple computerised random numbers, all patients were assigned to continue their SLOWB home training with the RESPeRATE^®^ device (Group 1) or not to continue this breathing exercise (Group 2) for the next 3 months. Between 3 and 6 months patients in group 1 were called by the same investigator (KL) weekly to ensure that patients attain their therapeutic breathing rate. At 6-month follow-up, the entire study cohort underwent comparable measurements including BP, HRV, 6MWT, echocardiography, CEPT and PWV.

### Statistical analysis

Results are expressed as means ± SD or percentage (%). Responses to SLOWB were analysed by repeated-measures One-way analysis of variance (ANOVA) comparing data between baseline and follow-up visits. Changes in variables between baseline and 3-month follow-up were analysed using a paired *t* test. The sample size analysis indicated that 12 subjects would have 80% power for a paired *t* test to detect an increase in peak RER of 0.08 at the level of significance 0.05 and an estimated standard deviation of 0.08. Our study revealed that 17 patients with a standard deviation of 0.09 and an increase in peak RER of 0.08 had 93% power. Statistical analysis was performed using SigmaPlot Version 13.0.0.83 (Systat Software, Inc. Leadtools, Dundas Software LTD. Reg. No. 775201235). A value of *P* < 0.05 was considered significant.

## Results

The analysis included a total of 21 patients (16 males, 5 females) with stable chronic HF diagnosed with diverse aetiology of HF including 9 patients with CAD and 12 patients without history of CAD. Past medical history included myocardial infarction (*n* = 8), paroxysmal atrial fibrillation (*n* = 3), persistent atrial fibrillation (*n* = 2), hypertension (*n* = 8), diabetes (*n* = 5) and chronic kidney disease with eGFR < 60 ml/min/1.73 m^2^ (*n* = 6). Therapeutic interventions included percutaneous coronary intervention (*n* = 6), coronary artery bypass graft (*n* = 3), ICD (*n* = 16) and cardiac resynchronization therapy (*n* = 7). None of the patients were current smokers.

Baseline demographic characteristics and drug distribution of the entire study cohort are demonstrated in Table 1. 21 patients with HFrEF in New York Heart Association classes I (*n* = 5), II (*n* = 13) and III (*n* = 3) had a mean LVEF of 23.9 ± 5.8%, age of 52 ± 17 years, BMI of 28 ± 4 kg/m^2^, waist of 100 ± 16 cm and hip of 103 ± 13 cm.

**Table 1:** Baseline characteristics of the study cohort

Parameter	Number (*n* = 21)
Age (years)	52 ± 17
BMI (kg/m^2^)	28 ± 4
Gender (males)	16 (76%)
ACEI	18 (86%)
ARB	3 (14%)
βeta-blockers	100%
Aldosterone antagonists	100%
Furosemide	11 (52%)
Torasemide	9 (43%)
Statins	14 (67%)
Aspirin	5 (47%)
Anticoagulants	12 (57%)
Haemoglobin (g/dl)	14.3 ± 1.1
BNP (pg/ml)	409.4 ± 419.6
Potassium (mmol/l)	4.4 ± 0.3
Sodium (mmol/l)	138.5 ± 2.5
Glucose (mmol/l)	108.4 ± 22.8
eGFR (ml/min/1.73 m^2^)	79.5 ± 31.3

Data are mean ± SD and/or percentage (%)

*ACEI* indicates angiotensin-converting enzyme inhibitor, *ARB* angiotensin II receptor blocker, *BNP* B-type natriuretic peptide

All patients were receiving stable dose of optimal multi-drug therapy which was kept unchanged for at least 6 weeks prior to study enrolment and was maintained (including drugs and dosage) over a 6-month study period. All patients were treated with beta-blockers (carvedilol, bisoprolol or metoprolol) and aldosterone antagonists (spironolactone or eplerenone). 18 patients were taking angiotensin-converting enzyme inhibitors (quinalapril, ramipril, enalapril or perindopril) and the remaining 3 patients were treated with angiotensin II receptor blockers (valsartan or telmisartan). Other drugs included amiodarone (*n* = 5), ivabradine (*n* = 4), trimetazidine (*n* = 3). A distribution of patients taking diuretics (furosemide, torasemide), statins (atorvastatin, rosuvastatin or simvastatin), aspirin and anticoagulants (warfarin, dabigatran or rivaroxaban) is shown in Table 1.

### Acute effects of SLOWB

Acute SLOWB reduced spontaneous respiration rate (18 ± 5 vs 8 ± 2 breaths/min, *P* < 0.001) and increased saturation (97 ± 2 vs 98 ± 2%, *P* = 0.01) but had no impact on beat-to-beat SBP (113 ± 7 vs 112 ± 9 mmHg, *P* = 0.12), DBP (88 ± 5 vs 88 ± 5 mmHg, *P* = 0.70) and HR (64 ± 8 vs 65 ± 7 bpm, *P* = 0.31).

Acute SLOWB normalized breathing pattern in HFrEF patients demonstrating daytime Cheyne–Stokes respiration.

### Long-term effects of SLOWB on respiration

On average, patients spent 170 ± 62 min per week in the therapeutic breathing zone. All patients reached final breathing rate of 6 ± 1 breaths/min at the end of the RESPeRATE session.

Breathing was synchronised with guiding tones in 61 ± 26% of time and the breathing sensor was able to detect breath in 89 ± 5%.

Spontaneous respiratory rate significantly decreased after 3 months of SLOWB home training (from 18 ± 5 to 14 ± 6 breaths/min, *P* < 0.05).

### Effects of SLOWB on BP

SLOWB significantly reduced office SBP (*P* < 0.001) but not central SBP, ambulatory daytime or nighttime SBP at 3-month follow-up (Table 2). Office DBP increased (*P* < 0.001) and nighttime DBP tended to increase (*P* < 0.07) but central and daytime DBP remained unchanged after 3 months of SLOWB home training (Table 2).

**Table 2:** Effects of slow breathing on blood pressure, arterial stiffness indices, 6-min walk test (6MWT) distance and echocardiographic parameters before and after 3 months for the entire cohort

Parameter	Baseline	3 M FU	*P* value
Office SBP (mmHg)	116 ± 11	100 ± 12	< *0.001*
Central SBP (mmHg)	119 ± 17	116 ± 17	*0.64*
Daytime SBP (mmHg)	112 ± 8	111 ± 9	*0.69*
Night-time SBP (mmHg)	100 ± 7	102 ± 9	*0.52*
Office DBP (mmHg)	72 ± 9	83 ± 9	< *0.001*
Central DBP(mmHg)	75 ± 8	73 ± 8	*0.36*
Daytime DBP (mmHg)	69 ± 8	69 ± 6	*0.96*
Night-time DBP (mmHg)	58 ± 6	61 ± 6	*0.07*
Office HR (mmHg)	72 ± 11	75 ± 12	*0.25*
PWV (m/s)	6.8 ± 1.7	7.0 ± 1.5	*0.30*
LVEF (%)	23.9 ± 5.8	25.9 ± 7.1	*0.10*
LVEDd (mm)	73.8 ± 9.7	75.3 ± 11.1	*0.45*
LVESd (mm)	63.2 ± 10.3	64.3 ± 11.8	*0.53*
LVEDV (ml)	288.9 ± 98.0	311.9 ± 100.3	*0.077*
LVESV (ml)	206.1 ± 86.3	224.8 ± 81.3	*0.18*
IVST (mm)	8.1 ± 1.6	8.6 ± 1.6	*0.01*
PWT (mm)	9.3 ± 1.2	9.5 ± 1.2	*0.45*
LA (mm)	47.9 ± 6.4	47.2 ± 10.4	*0.98*
LAA (cm^2^)	29.5 ± 6.9	30.5 ± 6.9	*0.26*
IVC (mm)	7.3 ± 5.5	5.4 ± 5.9	*0.21*
RAA (cm^2^)	22.6 ± 10.5	24.0 ± 11.3	*0.58*
RVID (mm)	38.5 ± 8.3	39.3 ± 9.1	*0.30*
TAPSE (mm)	19.4 ± 4.7	19.4 ± 3.6	*0.95*
RVSP (mmHg)	40.2 ± 14.5	38.5 ± 16.6	*0.25*
*E*/*E*′ ratio	10.6 ± 3.4	11.3 ± 4.5	*0.51*
6MWT (m)	422.7 ± 109.3	450.8 ± 92.4	*0.057*

Values expressed as mean ± SD

Data available on central BP (*n* = 16), ABPM (*n* = 18) and echocardiography (*n* = 20) at both visits

*SBP* indicates systolic blood pressure, *DBP* diastolic blood pressure, *HR* heart rate; bpm, beat per minute, *PWV* pulse wave velocity, *6MWT* 6-minute walk test, *LVEF* left ventricle ejection fraction, *LVEDd* LV end-diastole diameter, *LVESd* LV end-systole diameter, *LVEDV* LV end-diastole volume, *LVESV* LV end-systole volume, *IVST* intraventricular septum thickness, *PWT* posterior wall thickness, *LA* left atrium, *LAA* LA area, *IVC* inferior vena cava (on expiration), *RAA* right atrium area, *RVID* right ventricular internal diameter, *TAPSE* tricuspid annular plane systolic excursion, *RVSP* right ventricle systolic pressure, *E*/*E*′ the ratio of transmitral Doppler early filling velocity to tissue Doppler early diastolic mitral annual velocity

Between baseline, 3 and 6 months, daytime SBP (110 ± 7 vs 108 ± 11 vs 111 ± 5 mmHg, *P* = 0.67), daytime DBP (66 ± 7 vs 66 ± 6 vs 67 ± 6 mmHg, *P* = 0.68), nighttime SBP (100 ± 7 vs 100 ± 10 vs 101 ± 11 mmHg, *P* = 0.89) and nighttime DBP (57 ± 6 vs 58 ± 6 vs 57 ± 6 mmHg, *P* = 0.95) remained unchanged in group 1.

No changes in daytime SBP (111 ± 8 vs 115 ± 5 vs 115 ± 10 mmHg, *P* = 0.60), daytime DBP (70 ± 6 vs 73 ± 4 vs 72 ± 7 mmHg, *P* = 0.29), nighttime SBP (101 ± 8 vs 104 ± 8 vs 103 ± 5 mmHg, *P* = 0.62) and nighttime DBP (59 ± 6 vs 64 ± 6 vs 65 ± 7 mmHg, *P* = 0.17) were observed between baseline, 3- and 6-month follow-up, respectively in group 2.

### Effects of SLOWB on functional performance

6MWT tended to increase at 3-month follow-up for the entire study cohort (Table 2).

**Fig. 2:**
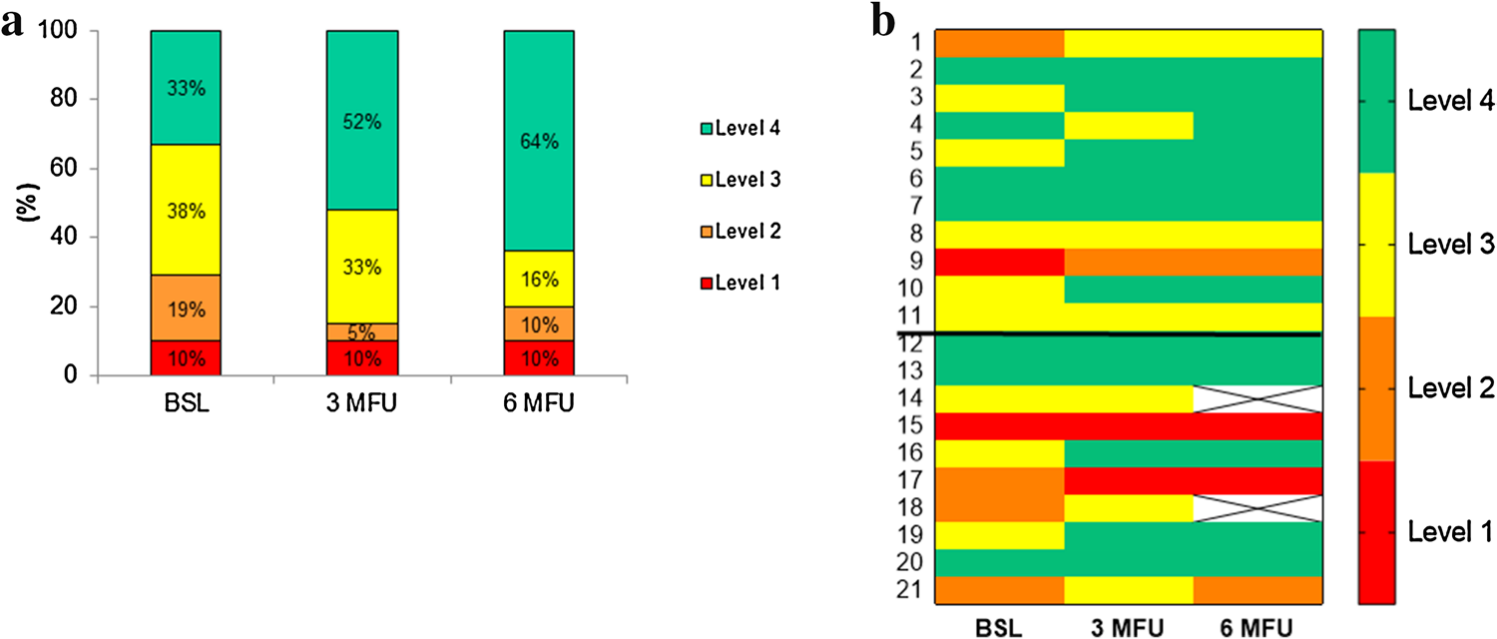
Proportion of patients with HFrEF in four performance levels based on the distance walked (**a**) and changes in individual patient data in four performance levels (**b**) at baseline, 3- and 6-month (M) follow-up (FU). Colours indicate four different performance levels; level 1 (the lowest performance level), level 4 (the highest performance level). Numbers from 1 to 11 indicate patients in group 1 assigned to 6-month SLOWB home training, numbers from 12 to 21 indicate patients in group 2 who underwent 3-month SLOWB home training and then a 3-month observation period (**b**). In group 2, two patients died (boxes marked without colour) between 3- and 6-month (M) follow-up (FU)

The study population was divided into four performance levels based on the distance walked as described previously [64]. At baseline, two patients were in level 1 (< 300 m), four patients in level 2 (300–374.9 m), eight patients in level 3 (375–449.9 m) and seven patients in level 4 (≥ 450 m). After 3 months, SLOWB substantially improved the levels of functional performance (Fig. 2a). At baseline, 29% (*n* = 6) of the study patients demonstrated level 1 and level 2 which was reduced by nearly half (15%) at 3-month follow-up (Fig. 2a). A number of patients at levels 4 category substantially increased from 33% at baseline, to 52% at 3 months, and 64% at 6-month follow-up (Fig. 2a).

Figure 2b demonstrates individual patient data moving across the four performance levels from baseline to 3- and 6-month follow-up in group 1 (numbers 1–11) and group 2 (numbers 12–21).

**Fig. 3:**
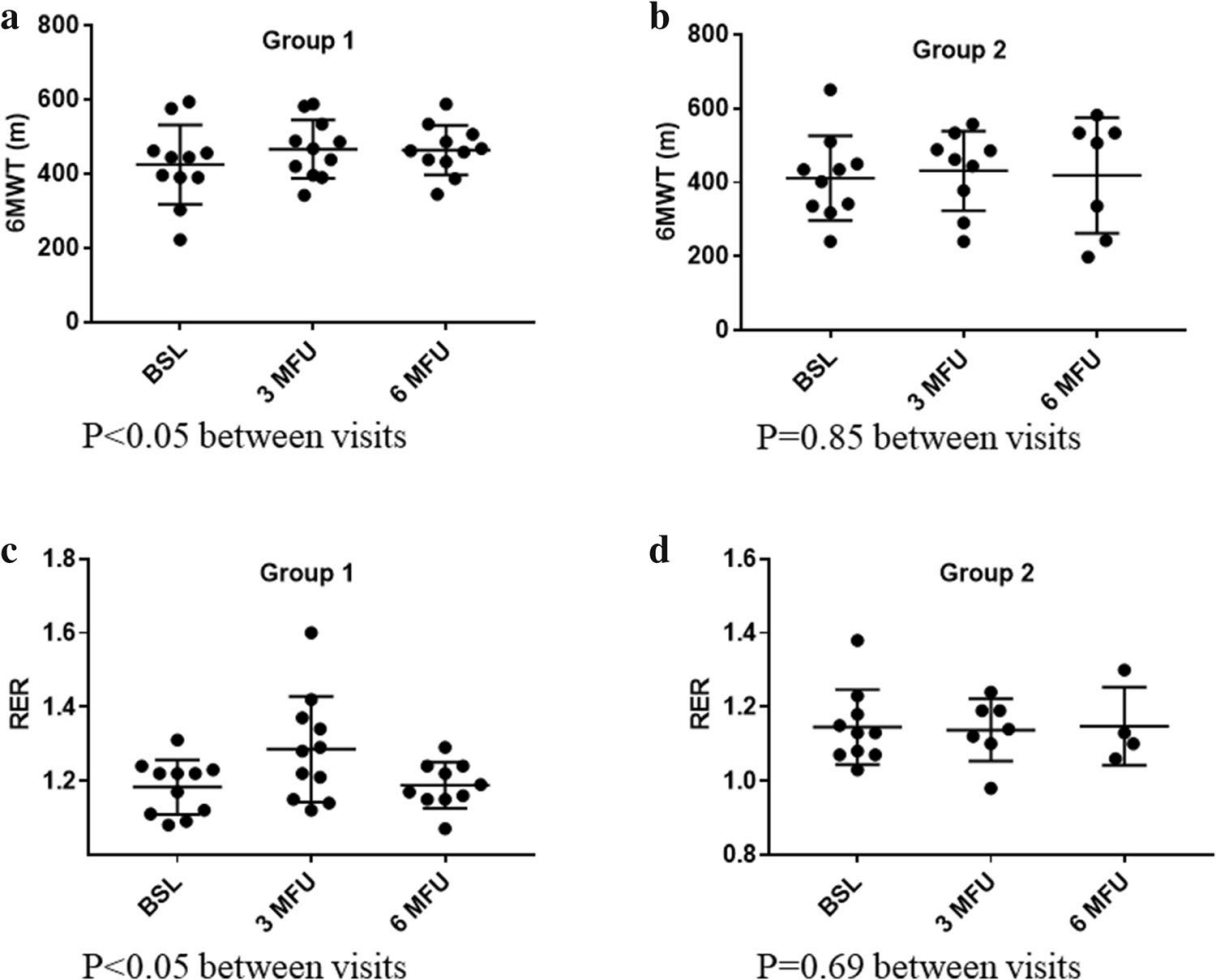
Effect of slow breathing on 6-min walk test in group 1 (**a**), group 2 (**b**) and respiratory exchange ratio (RER) in group 1 (**c**), group 2 (**d**) from baseline to 6 months (M) follow-up (FU). CPET was available in all patients at baseline, in 18 patients at 3 months (one erythema, one ICD intervention during 6MWT, one refusal) and in 14 patients at 6 months follow-up (two ICD interventions following 6MWT, one haemoptysis, one pulmonary infection, one refusal refused, two deaths)

Detailed analysis of the subgroups revealed a significant improvement in 6MWT in group 1 from baseline to 6-month follow-up (Fig. 3a) but not in group 2 (Fig. 3b).

SLOWB significantly increased peak RER from baseline to 3-month (1.16 ± 0.08 vs 1.23 ± 0.14, *P* < 0.05) follow-up. An improvement in peak RER was maintained out to 6 months in group 1 (Fig. 3c) but not in group 2 (Fig. 3d).

No changes were observed in P*V*O_2_ (14.3 ± 5.3 vs 13.4 ± 4.9 ml/kg/min, *P* = 0.14) and VE/*V*co_2_ slope (37.7 ± 7.4 vs 37.3 ± 8.7, *P* = 0.84) from baseline to 3-month follow-up.

### Effects of SLOWB on PWV and echocardiography

No significant changes in PWV and echocardiographic parameters were observed after 3 months of SLOWB (Table 2).

In group 1, between baseline and 6-month follow-up no significant changes were noted in LVEF (24.5 ± 5.6 vs 25.3 ± 6.8 vs 24.4 ± 7.7%, *P* = 0.75), EDV (285.5 ± 107.7 vs 305.4 ± 102.9 vs 310.8 ± 96.5 ml, *P* = 0.10) and ESV (201.9 ± 87.5 vs 215.6 ± 74.5 vs 223.9 ± 84.7 ml, *P* = 0.19), and in group 2 including LVEF (23.2 ± 6.3 vs 26.5 ± 7.6 vs 27.7 ± 9.8%, *P* = 0.30), EDV (292.7 ± 91.8 vs 319.8 ± 102.8 vs 279.9 ± 92.6 ml, *P* = 0.41) and ESV (210.7 ± 89.5 vs 236.0 ± 92.2 vs 207.7 ± 84.4 ml, *P* = 0.30).

### Effects of SLOWB on HRV

The final assessment of SLOWB effects on HRV was determined in nine patients from the study cohort who demonstrated no evidence of permanent cardiac pacing. There were no significant changes in SDNN total (126 ± 42 vs 124 ± 30 ms, *P* = 0.80) and RMSSD (42 ± 25 vs 52 ± 27 ms, *P* = 0.14) at 3-month follow-up. SLOWB improved SDNN/RMSSD ratio (3.26 ± 0.93 vs 2.51 ± 0.91, *P* < 0.05) at 3-month follow-up.

### Effects of SLOWB on CV outcomes

No patients experienced worsening, hospital admission or death for HF from baseline to 6-month follow-up (Group 1). However, following the 6-month SLOWB programme, three HF decompensations which required hospital admission and two HF worsening without hospitalization were observed. No deaths were observed within 1–2 years following the completion of the study. Two patients from this cohort underwent cardiac transplantation.

In group 2, between months 3 and 6 when patients were not continued SLOWB home training, two patients were admitted to hospital for HF decompensation and other two patients died (one death from HF-related decompensation, the second death from sudden cardiac arrest associated with epileptic seizures). Following the program completion, three additional hospitalizations due to HF decompensation and one HF worsening without hospital admission were noted. Within the group 2, two patients also experienced ICD interventions at 3-month follow-up, one during 6MWT, and another during CPET.

## Discussion

The present study is the first to demonstrate the 6-month effects of device-guided SLOWB home training in stable patients with severe HFrEF, all of whom received evidence-based treatment including optimal multi-drug therapy, implementable devices and surgical procedures, if required. The major findings are that (1) acute SLOWB improves oxygen saturation and (2) long-term SLOWB home training increases cardiorespiratory capacity, functional performance and vagal activity in high-risk patients with severe HFrEF irrespective of the underlying aetiology (i.e. ischemic and nonischemic HF). Neither acute nor long-term SLOWB had an impact on arterial BP and HR levels. No significant deterioration in echocardiographic parameters was noted over the course of the study. No complications including exacerbations, hospitalizations or deaths for HF were observed in patients undergoing a 6-month regular SLOWB home training. Slowing breathing rate with the use of RESPeRATE was well tolerated by the entire study cohort.

Altered reflex and neurohumoral factors underlying the development and progression of HF substantially contribute to the initiation and maintenance of augmented sympathetic activation. Impaired arterial baroreceptor function and increased chemoreflex sensitivity are of prognostic significance in HF, potentiating sympathetic activation in the setting of both ventricle systolic dysfunction and diminished inhibitory influences from reflex mechanisms [65]. The ability of the arterial and cardiopulmonary mechanoreceptors to exert tonic restraint on sympathetic outflow is also reduced in HF.

A decrease in breathing rate with concomitant prolonged expiration and inhalation can favourably influence autonomic CV regulation through the modulation of centrally-mediated neural reflexes. Previous acute studies demonstrated that slowing respiratory rate increases resting oxygen saturation, enhances respiratory muscles and pulmonary function (i.e. acute increase in peak oxygen consumption) leading to improved exercise performance, and reduced sensation of dyspnoea, and fatigue in chronic HF following 1 month of respiratory training [43]. This respiratory modulation induced by SLOWB has also led to reduced chemoreflex response [66] and highly significant increases in baroreflex sensitivity in chronic HF [67]. Data from the first randomized pilot study of a total of 24 patients with systolic chronic HF (mean LVEF 32 ± 6%) demonstrated the feasibility of device-guided RESPeRATE following 10 weeks of home training [46]. There were improvements in NYHA class, LVEF and quality of life, and a reduction in pulmonary pressure from baseline to 10-week follow-up [46]. An improvement in NYHA class and a reduction in breathlessness following a 4-week SLOWB training has also been found in a subset of HF patients (i.e. responders) compared to non-responders [48]. Findings from a recent study have demonstrated modest improvements in LVEF (31 ± 7 vs 32 ± 8%, *P* < 0.05), apnoea–hypopnea index (5.6 vs 5.4 events per hour, *P* < 0.05) and 6MWT (449.9 ± 122.7 vs 468.3 ± 121.9 m, *P* < 0.001) after 10–12 weeks of home passed breathing with the use of RESPeRATE in chronic HF [50].

Importantly, our findings are novel and indicate that the potential beneficial effects of SLOWB are not restricted to acute or short-term duration (12 weeks) of respiratory training but extend further to the longer term follow-up in patients with severe HFrEF as documented by mean LVEF (23.9 ± 5.8%), peak *V*O_2_ (14.3 ± 5.3 ml/kg/min) and VE/*V*co_2_ (37.7 ± 7.4). Acute reduction of spontaneous breathing rate was associated with significantly improved oxygen saturation. This increase in oxygen levels after SLOWB is likely to explain major improvements in functional performance levels assessed by 6MWT and cardiorespiratory capacity following home training at 3- and 6-month follow-up in our severe HFrEF cohort.

The 6MWT has been found a strong and independent predictor of increased morbidity and mortality in HF patients, with an increased risk inversely related to distance walked [64, 68]. This prognostic significance of 6MWT in predicting long-term death or hospitalization rates for HF was independent of LVEF and NYHA class [64].

There is evidence to suggest that in HF patients, NYHA classification system is subjective and poorly reproducible, particularly in differentiating between patients belonging to class II and class III [69]. Moreover, 6MWT weakly correlates with LVEF and NYHA class II which includes patients with moderate impairment of HF and a wide range of walking distance [64]. Convincingly, 6MWT has been clearly linked to the extremes of the NYHA classification (i.e. longer walking distance evident for patients in NYHA class 1, shorter walking distance for patients in NYHA class IV) [64]. Our study corroborates this notion and demonstrates a significant but inverse relationship between 6MWT and NYHA class (*r* = − 0.81, *P* < 0.0001) and a lack of correlation between 6MWT and LVEF (*r* = 0.18, *P* = 0.45).

In this context, our findings are supportive in demonstrating substantial improvements in the performance levels (based on the distance walked) following SLOWB home training in patients with severe HFrEF. While it appears that all patients with severe HFrEF may benefit from SLOWB therapy irrespective of baseline functional capacity (Fig. 2b), it appears that this favourable effect on performance level remains sustained and extends further if paced breathing is continued over the longer term in addition to optimal medical therapy for HF.

A further novel finding achieved with SLOWB is an improvement in cardiopulmonary exercise testing in our severe HFrEF patients as demonstrated by an increase in peak RER. Amongst parameters derived from cardiopulmonary exercise testing in HF, peak RER is considered as an indicator of a patient’s level of maximal exertion, good indicator of subject effort and intrasubject effort during serial testing (i.e. pre- and postintervention), and that is independent of patient characteristics (i.e. age, gender, fitness, and disease state) [70]. An improvement in peak RER remained significant at 6 months of regular SLOWB when compared to 3-month period of therapeutic breathing only. Given lacking supportive evidence for the use of 6MWT as a prognostic marker in alternative to or in conjunction with CPET-derived variables [71], an improvement in peak RER after SLOWB appears to be clinically relevant.

Our HFrEF patients demonstrated no significant changes in central, ambulatory daytime or nighttime BP profile and reported no incidence of dizziness or orthostatic hypotension indicating the safety of therapeutic SLOWB home training. This is clinically important as the RESPeRATE provides the possibility of continuing life-saving pharmacological treatment without the risk of ceasing medication during SLOWB therapies.

Another important observation from this study is the absence of significant deterioration in echocardiographic parameters including LVEF, EDV and ESV following SLOWB home training suggesting no further progression of severe HFrEF. Moreover, in line with previous findings we observed an improvement in LVEF by 2 ± 5% at 3-month follow-up [50]. The effects on PWV were not observed in our study indicating no BP-dependent or BP-independent effects of SLOWB on large artery remodeling.

An interesting observation which may deserve further investigation in appropriately sized clinical trials is changes in neural regulation of heart rhythm produced by deep and slow breathing. A decrease in SDNN/RMSSD ratio following SLOWB suggests a shift toward increased parasympathetic activity which theoretically may lead to improved patient outcomes.

Finally, given the link between Cheyne–Stokes respiration and increased mortality in ambulatory patients with severe HF [72], a normalization of breathing pattern during a 15-min duration of paced device-guided SLOWB exercise is another important finding which deserves further investigation.

Unlike previous studies on SLOWB in HF in which patients were asked immediately to reduce their breathing rate to 6 breaths/min, our patients were asked to breathe effortlessly and gradually at home twice daily aiming at slowing breathing rate below 10 breaths/min which prevented forced breathing, patient discomfort or associated emotional stress. Indeed, the final breathing rate achieved in this study was 6 ± 1 breaths/min and no patients resigned from using the RESPeRATE over the study duration. Furthermore, in addition to improved functional capacity, a subjective reduction of dyspnoea reported by our severe HFrEF patients had a positive impact on patient adherence in continuing their SLOWB home training.

Most importantly, no patients experienced exacerbation of their pre-existing severe HFrEF condition, no hospitalizations or deaths for HF occurred in the group assigned to 6-month SLOWB home intervention.

### Study limitations

The relatively modest number of patients limits our ability to compare the effects of time and treatment between 3- and 6-month follow-up among both study arms. Nevertheless, this study is the first which examined the 6-month effects of SLOWB on prognostic clinical variables in patients with severe HFrEF, all of whom received optimal evidence-based treatment options. In this study, medication remained unchanged at follow-up which allowed for an adequate determination of intrasubject effectiveness of SLOWB on BP, HR, 6MWT, echocardiography, CPET and HRV from baseline to 6-month follow-up with the use of repeated measures analysis. Given the high-risk patient cohort, all these tests were performed during hospitalization at three different study visits. Furthermore, this modest number of patients allowed for close monitoring of compliance to SLOWB therapy by weekly phone call to each patient and information derived from the device memory including accumulated minutes of therapeutic breathing rate, initial and final breathing frequency and percentage of synchronization respiratory rate with guiding tones, and breath detection. While we observed no HF-associated adverse events during 6 months of SLOWB home training, this requires further investigation in future clinical trials.

In conclusion, our study suggests that SLOWB, with the use of RESPeRATE, if applied adequately (i.e. therapeutic breathing rate < 10 breaths/min achieved daily and synchronised with guiding tones as recommended in the RESPeRATE’s built-in memory stores) can slow the progression of severe HFrEF in patients on optimal medical therapy, irrespective of the underlying aetiology of HF. The objective evidence for the benefits associated with SLOWB indicates that an extensive use of this intervention as an add-on therapy to the state-of-the-art management for HF may have a substantial impact on patient care and clinical outcomes, which should be further confirmed through larger clinical trials with longer term observations.
